# Newly enacted mental health law in Bangladesh

**DOI:** 10.1192/bji.2021.1

**Published:** 2021-11

**Authors:** Mohammad Ershadul Karim, Sabuj Shaikh

**Affiliations:** 1 PhD, Senior Lecturer, Faculty of Law, University of Malaya, Kuala Lumpur, Malaysia. Email: ershadulkarim@um.edu.my; 2 Barrister (Lincoln's Inn), Junior Professional, Rule of Law Programme, Deutsche Gesellschaft für Internationale Zusammenarbeit (GIZ) GmbH, Bangladesh

**Keywords:** Human rights, mental health law, mental health in Bangladesh, health and human rights, mental illness

## Abstract

Mental health problems are almost ignored in Bangladesh, one of the most densely populated countries in the world. The lack of overall health literacy and human resources due to an ineffectively updated legal and regulatory framework, coupled with very limited but misused budget allocation, are some of the factors responsible for this. The country's Constitution recognises the importance of public health and stipulates the improvement of public health as an important primary duty of the state. Nevertheless, it is often compromised or neglected in favour of other socioeconomic development priorities. The Lunacy Act 1912 was recently repealed and substituted by the Mental Health Act 2018 to fill in various gaps in mental health law. This is a welcome development, but there remain limitations and scope for further improvement. We highlight some important provisions of this newly enacted law, identify some limitations and propose some issues for consideration in future policy reform.

## Background

In Bangladesh, one of the most densely populated countries in the world, mental health problems are almost ignored owing to the lack of overall health literacy, although some positive recent developments can be noticed. One study found signs of mental disorder prevalent among 6.5–31.0% of the adult population and 13.4–22.9% of children.^1^ Although the human right to health, more specifically public health, is recognised in the Constitution of Bangladesh 1972, this generally receives less priority in the context of ongoing overall development initiatives.^2^

At the time of independence in 1971, Bangladesh inherited only one specialised mental health hospital^3^ and a century-old statute, the Lunacy Act 1912. This law, enacted in a different context, was used to govern the overall metal health legal regime until recently. It was not mental health legislation in the true sense and had long been criticised for being ‘archaic and obsolete’, for the Act focused more on segregation and detention of people with mental illness, ignoring their well-being and rehabilitation within the society.^4^ Therefore, there has long been a demand for the enactment of a new mental health law incorporating human rights-based approaches following international best practices. Simultaneously, most health-related national legislation had become too outdated to meet the demands of the time.^2^ Against this backdrop, the National Health Policy 2011 was framed, in which ‘health’ was recognised as complete physical, mental and social well-being, and the increased mental health problems resulting from urbanisation have been acknowledged. Consequently, the Mental Health Act 2018 (MHA 2018) was enacted with the aims of providing healthcare services and protecting the right to property and rehabilitation and the overall welfare of people with mental disorders or illnesses. This paper highlights some important provisions of this law and identifies some limitations for consideration in future policy reform.

## The Mental Health Act 2018: an overview

The MHA 2018, a special law having overriding effects over other existing laws, contains provisions on four major issues: (a) establishment and supervision of mental health hospitals and rehabilitation centres; (b) assessment, admission and treatment of mental health patients; (c) judicial examination of mental health and determination of mental capacity; and (d) guardianship of the person and property of such patients. The MHA 2018 defines some relevant terms, such as consent to treatment, mental health, mental disorder and mental illness. The term ‘consent to treatment’ is loosely defined, and a subtle distinction between mental illness and mental disorder is made. ‘Mental disorder’ is defined as conditions, including mental disability, drug addiction and any other clinically recognised mental conditions, that, being connected with a person's body and/or mind, hinder their normal living, whereas ‘mental illness’ is defined as a form of mental illness other than mental disability or drug addiction.

The MHA 2018 lays down a more detailed process and procedure for assessment, admission and medical treatment of those with mental disorders. For assessment and admission, mental health patients are classified as: voluntarily admitted patients, non-protesting patients and unwilling patients. Adults with mental illness may be admitted to hospitals voluntarily, but for minors, the consent of their guardians (i.e. parents) or relatives is required. Voluntarily admitted patients may seek release unless their admission status is changed and they are subjected to involuntary treatment. For a non-protesting patient, someone who has a mental illness but is incapable of giving an opinion about their admission or treatment in a mental hospital, a guardian or relative may apply for admission and the patient can be admitted on the basis of an examination conducted by a responsible medical practitioner. The involuntary admission and treatment of unwilling patients, with or without an application by a guardian/relative or a police officer, is permitted once a psychiatrist, after considering the nature and severity of the person's illness and relevant matters, certifies mental illness or mental disorder even though the intended person denies.

The MHA 2018 provides for the establishment of a Mental Health Review and Monitoring Committee (MHRMC) at district level to review the rationality of admission and treatment of each psychiatric patient. The MHRMC is required to review voluntarily admitted adults every 15 days and children every 7 days, and non-protesting patients every 28 days. Subject to periodic review by the MHRMC and recommendation of a responsible medical officer or a psychiatrist, the period of involuntary admission for psychiatric treatment can range between 3 and 28 days, and can be extended up to 180 days or more, if needed. The law provides for the admission and treatment of patients who do not have guardians/relatives or shelter to a social welfare institution or rehabilitation centre on discharge from hospital.

The MHA 2018 provides that the father or mother is the guardian of the person and property of someone with mental illness. In their absence, other persons, including relatives, may act as guardian. A person's mental capacity can lawfully be assessed through judicial intervention with the appropriate court order to settle guardianship and property management issues. A guardian or manager of property shall be criminally liable for negligence in performing the assigned duties and responsibilities.

The MHA 2018 empowers the government to establish separate mental health units in the country's medical college hospitals and all district-level government hospitals, and permits the operation of related rehabilitation centres by private entities. This is an appreciable move, as government-run public health facilities are inadequate, resulting from inadequate and misused budget allocation, and available facilities are clustered in big cities.^1^

## The missing points

The MHA 2018 is an important development in the promotion, protection and preservation of mental health rights in Bangladesh, but there are some limitations. First, although the Act recognises the importance of mental health patients’ rights to health, property, dignity, education, etc., it does not contain an exhaustive list of such rights. Besides, there is no provision on the infringement of these rights when the treatment of such patients is difficult owing to an unfavourable or adverse social environment. Although the rights of mentally disabled persons are recognised through the Rights and Protection of Persons with Disabilities Act 2013, subsidiary legislation is to be framed to protect the rights of persons suffering from mental illnesses that fall short of mental disability. Second, issues of patient's confidentiality and associated accountability of medical practitioners for failure to maintain confidentiality are not included in detail.^5^ Third, the issue of social rehabilitation and integration of mental health patients through community-based treatment, which is believed to be more effective than institutional treatment, is not recognised.^5^ Fourth, the law does not provide for the adequate training of mental healthcare service providers about patients’ human rights.

The inclusion of provisions on criminal liability for providing a false certificate of mental illness has raised some concerns among the service providers. When Bangladesh does not have even one psychiatrist per million inhabitants, some believe that such legal provision may discourage psychiatrists from examining and treating patients, thus barring them access to mental healthcare services, for fear of punishment. Nevertheless, this is justifiable in the context of a country in which false certificates can be doctored easily which has serious implications for the exercise of property rights, inheritance, guardianship, custody and other marital rights, criminal liability, etc.^6,7^

## The way forward

Bangladesh has an enormous burden of mental health problems and significantly disproportionate mental health facilities. During the COVID-19 pandemic, which has compelled people to stay at home more, an important segment of citizens has realised the great importance of mental healthcare. Although around 16% of the country's population suffers from some sort of mental disorder, only 10% of those individuals receive treatment.^5^ Others remain untreated owing to social stigma or medical expenses, about 70% of which are borne personally by the patients or their relatives.^8^ Only 6% of the national budget, which is less than 1% of the country's gross domestic product, is allocated to the healthcare sector, and of this, less than 1% is allocated to mental healthcare and services.^8^ Unfortunately, this small allocation is not even materialised properly because of corruption: healthcare is recognised as one of the most corrupted government sectors.

Ensuring accessible and close-to-community mental healthcare services may not be possible in the near future in Bangladesh for both socioeconomic and cultural reasons. Nevertheless, the government should set the priority to establish mental health units in the medical colleges and district level government hospitals as soon as possible, adhering to the provisions of the law. The newly enacted MHA 2018 is a welcome development; however, much is still to be done. In particular, Bangladesh urgently needs the framing of associated subsidiary legislation and mental health policy detailing provisions regarding informed consent and the rights of mental health patients (following a human rights-based approach). It also needs to build service capacity and develop social awareness about the importance of mental healthcare and a healthy family life, social inclusion and community. Such actions and policies would do much to create a mentally sound and healthy nation for the future.
